# How can we change medical students’ perceptions of a career in family medicine? Marketing or substance?

**DOI:** 10.1186/s13584-018-0248-6

**Published:** 2018-08-25

**Authors:** Amnon Lahad, Andrew Bazemore, Davorina Petek, William R. Phillips, Dan Merenstein

**Affiliations:** 1 Departments of Family Medicine, Hebrew University & Clalit Health Services, Jerusalem, Israel; 2 Faculty of Medicine, The Hebrew University of Jerusalem, Ein Kerem, P.O. Box 12271, 9112102 Jerusalem, Israel; 3 Robert Graham Center Policy Studies in Family Medicine & Primary Care, 1133 Connecticut Ave, NW Ste#1100, Washington, DC, 20036 USA; 4 Department of Family medicine Faculty of Medicine, University of Ljubljana, Poljanski nasip 58, 1000 Ljubljan, Slovenia; 5 Family Medicine, University of Washington, Box 356390, Seattle, WA 98195-6390 USA; 6 Department of Human Science, Research Programs Family Medicine, Georgetown University Medical Center, School of Nursing and Health Studies, Building D 240, 4000 Reservoir Road, NW, Washington, DC, 20007 USA

**Keywords:** Family medicine, Medical education, Medical students, Career choice, Medical specialty

## Abstract

Family Medicine (FM) is the care of unselected patients with undifferentiated problems in the settings where people need care in our communities. It is intellectually challenging, providing breadth and depth unparalleled in other areas of medical practice. In one survey only 19% of Israeli students reported being interested in FM. Students interested in FM had greater interest in bedside and direct long-term patient care. Students not planning FM residency training had preconceived notions that the discipline had lower academic opportunities and prestige. What can be done to increase student interest in careers in FM?

This commentary includes perspectives of family practice leaders from several countries:

The problem isn’t the students it is the scope of practice and expectations both of which can and should change if FM in Israel wants to stay viable. The scope of FM should be broadened to include more procedures and new technologies. This may also increase the earning potential of Family Practitioners (FPs). Payment policy and credentialing barriers should be change to expand scope of practice and allow FPs to practice at the full extent of their training.

FM should offer clear professional horizon with potential for many sub-specialties and areas of focus. The Israeli HMOs, the Ministry of Health and the Israeli Association of FM should invest heavily in building academic departments of FM and promoting research. This will enhance the image of FM in the eyes of the students, the profession and the public.

The clinical work environment should be improved by reducing bureaucratic assignments, such as issuing certifications, dealing with quality measurements and renewing chronic prescriptions. Much of this work can be done by nurse practitioners (NPs) working as part of an FP-led team. These NPs can also take care of patients with limited complaints to make the work of the FP more challenging and attractive.

Training must include opportunities to develop longitudinal relationships with patients and families across problems and over time. It is these relationships that add value to the process of care, improve patient outcomes and provide meaning to sustain clinical careers that meet the needs of patients and communities.

The article by Naimir, et al. in a recent issue of *Israel Journal of Health Policy Research* identified a major problem of the Israeli medical care system: Why do Israeli medical school graduates rarely choose careers as Family Physicians (FP)? In their survey, only 19% of students reported being interested in Family Medicine (FM).

Like surveys in other countries [1–4], students interested in FM had greater interest in bedside care with direct long-term patient relationships and also had a sense of mission as a physician.

Like several other surveys [1, 2, 4], they found that female students tend to choose FM at higher rate than male students do. Students not planning FM residency training have preconceived notions that the discipline has lower academic opportunities and prestige. Even though their study lacked the power to deeply examine this problem and did not represent all Israeli graduates or all young physicians seeking a residency, it is unique in its view that this problem is a marketing issue.

In 2016, the Brandman Research Institute and the Israeli Association of FM conducted focus groups on “choosing a residency” with medical students in their last 2 years from all 5 medical schools in Israel [5]. Interest in FM was below surgery, GYN and internal medicine. The students felt that FPs can have reasonable quality of life, can have positive impacts on the lives of their patients and can practice medicine in the “old style”. At the same time, students felt FM provided limited ability for professional growth and high income. They also felt the clinical work environment of FM is less competitive compared to the hospital, but lacked the teamwork that existed on hospital wards. This is in some accordance with the OECD reviews of *health care quality – Israel*, reporting that care management and co-ordination for chronic conditions is still largely a physician-led activity in Israel, contrary to the fact that physicians prefer higher rates of nurse involvement in patient care [6]. The main disadvantages of FM reported by these students were the low prestige among colleagues and the public, lack of “action” and lack of intellectual challenge and academic opportunities. Another serious disadvantage was the dense scheduling in clinics, with less than 10 min per patient. The students also felt the short exposure to FM during medical school was a problem.

What can be done to increase student interest in specialty training and careers in FM? We gathered responses from several leading FPs in the US and Europe:

*Andrew Bazemore MD MPH, Director, Robert Graham Center Policy Studies in Family Medicine & Primary Care, USA:*

Research by Naimir and the Brandman Institute highlights several important perception challenges impeding to student entry into Family Medicine, each worth addressing in turn:

1. *FM is less interesting than other specialties*: The range of services, settings, and conditions Family Physicians are trained to care for is unrivaled, but payment policy and credentialing barriers limit scope of practice in many developed nations, particularly as specialists grow in number and authority [7], to the detriment of health system costs, quality, access and equity. The time is ripe, in this 40th anniversary year of the Declaration of Alma Ata, to engage policymakers in a better understanding of Primary Health Care [8], and why payment and practice policy should allow FPs to practice at the full extent of their training. Specific efforts should be made in training programs and practice to expand the range of procedures regularly performed by FPs, expand their capacity to collaborate with the public and community health workforce and its efforts to address social determinants of health, and create post-graduate certifications in specialty areas (geriatrics, sports med, research, women’s health, etc.)
2. *Family Physicians have limited ability for professional growth and high income*: From my visit to Israel and awareness of comparative systems literature, I sense that the primary care-specialty salary gap in Israel is lower than in most higher income nations, and that countering this perception will rely more on improving FP autonomy in practice and scope of practice. In addition to payment reforms and practice transformation noted above, students considering FM might find inspiration in disruptive innovations in primary care practice and delivery that promote professional growth, self-esteem, and autonomy going on around the world. Examples from the U.S. include Direct Primary Care [9], where providers have used individual and whole employer retainer fees to shrink their panel size, grow IT innovations, allow around the clock accessibility, and improve patient and provider satisfaction. Efforts should also include intentional development of FM leaders within Israel’s four health system payors, its hospital delivery systems, and national health ministry. I learned during my visit to Israel that there is a number or Israeli FPs already serving in leadership managerial positions, which should be marketed to medical students.
3. *FM offers less prestige and is not academic enough*: In training, creating primary care leadership and research tracks may help counter this perception (https://www.hopkinsmedicine.org/office-of-johns-hopkins-physicians/best-practice-news/primary-care-leadership-track-rolls-out, https://medschool.duke.edu/education/degree-programs-andadmissions/primary-care-leadership-track), and help FPs to grow as leaders in medical education and research. Growth of national practice-based research networks to capture the power of primary care practices in the national research enterprise is also critical to growing awareness of develop certificate programs or fellowships in med-ed/primary care research.
4. *There is an overall lack of interest in FM*: One way to increase current incentives or create new ones for people to enter FM would be the creation of Family Medicine Interest Groups [FMIG] in medical schools. The Doctor of Medicine -Military Medical Track - “Tzameret” may also be an opportunity to increase recruitment to primary care. Most graduates of this track will serve as military Primary Care (PC) doctors and will need a greater understanding of PC from day one of their service.

Finally, Israel could look to campaigns like “Health is Primary” [http://www.healthisprimary.org/] and “Family Medicine for America’s Health” (FMAH) [10] to as ideas for a marketing approach to promoting FM nationally. Since FM is not prestigious enough also in the public’s eye, you should start a national campaign similar to the USA ‘Health is Primary’. It would help to have more family doctors featured in the media. For example, you could work with news outlets to have family doctors be featured columnists.

*Dan Merenstein, MD. Director of Research Programs Family Medicine, Georgetown University Medical Center, USA:*

There are significant differences between what it means to be a FP in Israel and the US. There are many reasons we have problems recruiting in the US, a lot having to do with money and prestige, both clearly intertwined, like the results of the survey in Israel. But FPs in the US are known to do many procedures, have hectic practices and one of the reasons students stay away is due to there being so much going on, that it is intimidating. FPs in Israel sounds more like specialists in General Internal Medicine (GIM) in the US, but don’t have the inpatient experience GIM doctors in the US get to experience. It seems to me it would be important to change the scope of what FPs are doing, empower them to do more outpatient procedures and find aspects of care they love. In the US, FPs find a niche without formal training, where one doctor will do a lot of procedures, another a lot of musculosketal, one research and another women’s health. There are a lot of reasons that young physicians may decide that FM isn’t for them but lack of procedures, boredom and little action shouldn’t be any of them. If they are, it seems like the problem isn’t the students but the scope of practice and expectations, both of which can and should change if FM in Israel wants to stay viable.

*Davorina Petek, MD, PhD, Department of Family Medicine, Faculty of Medicine, University of Ljubljana, Slovenia:*

Slovenia has a low number of physicians per capita (2.4/1000), but is highly oriented to PC. FPs have on the average of 1800–1900 patients on their lists, with 40–50 consultations per day working as gatekeepers.

The government has accepted new standards for FM, with 1500 patients per FP. To reach this goal the number of FPs should be substantially increased in coming years.

There is a constant effort to increase the number of available training positions for FPs, which is a joint decision of the Medical Chamber and the Ministry of Health, and to attract young medical graduates to choose FM for their career. Moreover, good working conditions and lower burden of everyday work should also be achieved.

Over the last decade, the interest of graduates in specializing in FM has substantially increased. This can be attributed to an increase in the social and professional reputation of FM, which has become an academic discipline, and to careful planning of undergraduate education.

It is important that the students meet with FMs while visiting family practices in their first years, where they have contact with patients and join FPs for home visits. Later on, they can choose between several courses, such as “The Motivational Interview” or “Rural Medicine”. Research oriented students can choose the course “Research in FM” and take part in the research projects.

The students have to meet FM in its attractive form, so a 6-week course in FM that takes place in the last year of medical school is practically oriented and students spend most of the time in FM practices. Students who choose FM as a career option appreciate long-term patient-doctor relationships, complex problem solving and dealing with psychological and physical problems at the same time. They can get acquainted with these aspects of FM working closely with a mentor who generally presents a very good role model [11]. We encourage students to take part in competing university calls for student research awards, showing students that FM is not a simple discipline devoid of challenges, and that they will have good options for academic careers in FM if they want. Successful students present their projects at international conferences with the financial help of the Department of FM.

*William R. Phillips, MD, MPH Department of Family Medicine, University of Washington, Seattle, WA. USA (Emeritus):*

FM is the care of unselected patients with undifferentiated problems in the settings where people need care in our communities. It is intellectually challenging, providing breadth and depth unparalleled in other areas of medical practice. Medical specialists, including most teachers in academic medical centers with their skewed perspectives on patients and their problems, fail to understand the essence of generalist care. They do medical students a disservice by passing along ill-informed caricatures of general practice that lead many students to choose highly paid careers in limited areas of practice. These choices may not meet the needs of the learners, patients or our communities.

Strategies to attract more students to fulfilling careers in FM include recruiting students oriented toward whole patient care and community service, training them in curricula that include comprehensive care in community settings from start to finish, and offering them meaningful experience with real FPs, taking care of real patients, with real problems. Students should be required to make meaningful contributions to PC teams that provide acute, chronic and preventive care to patients of all ages, regardless of sex, organ system or disease. In this setting, role model FPs demonstrate high levels of synthetic thinking to solve complex clinical problems. They practice the full variety of diagnostic and therapeutic procedures dictated by patient needs. The intellectual challenges of managing multiple problems in real-world settings expose needs for evidence that stimulate curiosity for research and scholarship. Limitations of current science and the constant challenge of improving practice stimulate reflective practice, professional growth and personal fulfillment. Training must include opportunities to develop longitudinal relationships with patients and families across problems and over time. It is these relationships that add value to the process of care, improve patient outcomes and provide meaning to sustain clinical careers that meet the needs of patients and communities.

*Amnon Lahad, MD, MPH, Head, Departments of Family Medicine, Hebrew University & Clalit Health Services, Jerusalem, Israel. Chairman of the Israeli National Council for the Health of the Community:*

While there is a discrepancy between the image of FM and the real life of a FP, the main problem with recruiting young MDs to FM residency is not the marketing but the real content of the FM specialty. We must increase the attractiveness of FM by making several important changes:

- FM should offer a clear professional horizon with potential for many sub-specialties and areas of focus, including sub-specialty of internal medicine in the community [e.g. endocrinology, rheumatology, clinical pharmacology, pain management, diabetic mellitus, medical education, CBT].
- The Israeli HMOs, the Schools of Medicine, the Ministry of Health and the Israeli Association of Family Medicine should invest heavily in building academic departments of FM and promoting research. This will enhance the image of FM in the eyes of the public and profession.
- The clinical work environment should be improved by reducing bureaucratic assignments, such as issuing certifications, dealing with quality measurements and renewing chronic prescriptions. Much of this work can be done by nurse practitioners (NPs) working as part of a FP-led team. These NPs can also take care of patients with limited complaints to make the work of the FP more challenging and attractive (https://www.health.gov.il/PublicationsFiles/NURSE_23012018.pdf, [12]).
- The scope of FM should be broadened to include more procedures and new technologies. This may also increase the earning potential of FPs.
- Medical schools should strengthen training in FM, in ways demonstrated to influence the career choices of graduates, [13] mainly by providing early exposure to FM and particularly to academic FM. This will require fundamental changes in Israel’s schools of medicine.

## Conclusions

Challenges to recruiting young MDs to FM residencies in Israel are multifactorial and include the structure of medical training and practice. Factors include inadequate early exposure of medical students, a limited scope of practice and poor marketing. Essential changes include creating attractive professional horizons, promoting research and increasing clinical team work.
